# Big Public Welfare Programme

**Published:** 1920-07-17

**Authors:** 


### Big Public Welfare Programme.

### The Royal Sanitary Institute Congress.

The Congress of the Royal Sanitary Institute, which is to assemble in Birmingham on Monday and continue throughout the week under the Presidency of Viscount Astor, will have under its consideration a very wide range of problems affecting public health and well-being.

The programme begins appropriately with matters affecting the birth-rate, and contains some sixty other subjects upon which information and discussion are desirable. It includes matters affecting child welfare, housing, industrial hygiene, and ranges from engineering work, such as road pavements, to the father's responsibility in relation to the home, and the care of the lonely and aged poor. The sections are as follows:—

*Sanitary Science and Preventive Medicine.*—Among the subjects to be discussed are mentally defectives, production of clean milk, the falling birth-rate, malaria, disinfectants, influenza, pneumonia, and allied epidemics.

*Hygiene of Maternity and Child Welfare.*—The still-birth problem, teaching of sex hygiene, infant welfare work, and recreation of children.

*Engineering and Architecture.*—Housing, reconstruction of slums, school buildings, house drainage, and river pollution.

*Personal and Domestic Hygiene.*—Choice and storage of foods, child hygiene, and physical culture.

*Industrial Hygiene.*—Industrial efficiency and fatigue, welfare work in factories, and the health of seamen.

There are seven technical conferences of sanitary authorities and their officers arranged in connection with the Congress. There is a special conference on the work of local authorities, and the work of the Birmingham Corporation departments is to be practically illustrated at the Health Exhibition which is arranged in connection with the Congress. Among the delegates several public departments in England, Scotland, and the Overseas Dominions, including the Ministry of Health, are to be represented, and delegates are also being sent by Denmark, France, Holland, Sweden, and, in addition, some 600 delegates from English boroughs.

Twenty years ago the Royal Sanitary Institute held a very successful meeting in Birmingham, and since that date the importance of health as a national asset is more generally understood, and the adjustment of the many factors that make for efficiency should be advanced by this large and representative meeting of experts.

